# Prognostic value of Annexin A3 in human colorectal cancer and its correlation with hypoxia-inducible factor-1α

**DOI:** 10.3892/ol.2013.1620

**Published:** 2013-10-11

**Authors:** YONG-QIU XIE, DI FU, ZHENG-HUA HE, QING-DONG TAN

**Affiliations:** Department of Anesthesiology, Xiangya Hospital of Central-South University, Changsha, Hunan 410008, P.R. China

**Keywords:** colorectal cancer, Annexin A3, immunohistochemistry, hypoxia-inducible factor-1α, prognosis

## Abstract

Annexins are a family of intracellular proteins that bind membrane phospholipids in a Ca^2+^ concentration-dependent manner and are involved in cellular processes, including apoptosis, proliferation and differentiation. Hypoxia-inducible factor-1α (HIF-1α) has been hypothesized to be critical in the angiogenesis of tumors. We hypothesized that Annexin A3, a member of the Annexin family, and HIF-1α may be associated with each other in colorectal cancer. The expression of Annexin A3 and HIF-1α in 60 colorectal cancer tissues was assessed by immunohistochemistry to statistically analyze the association between the clinicopathological features and survival of these cases. In the present study, 65 and 47% of colorectal cancer specimens were found to show Annexin A3 and HIF-1α immunoreactivity, respectively. Annexin A3 expression was found to significantly correlate with tumor size and Dukes’ stage (all P<0.05). Furthermore, Annexin A3 and HIF-1α protein expression exhibited a similar pattern in these samples, and their expression was found to correlate with poor survival in colorectal cancer patients. The results of the current study indicated for the first time that the increased expression of Annexin A3 in colorectal cancer correlates significantly with tumor growth and poor prognosis. Furthermore, Annexin A3 has been found to correlate with HIF-1α expression. These observations highlight an improved understanding of the carcinogenesis of colorectal cancer.

## Introduction

Colorectal cancer is a common malignant tumor of the gastrointestinal tract, constituting ~10% of new colorectal cancer cases each year. Despite major breakthroughs in the treatment of colorectal cancer, 40–50% of patients are likely to develop local or distant tumor recurrences, and survival rates are poor (1). Therefore, the development of a novel therapeutic target is required, with the aim of increasing the survival rates of colorectal cancer patients.

Ca^2+^-dependent phospholipid-binding proteins, Annexins, are involved in cellular processes, including apoptosis, proliferation and differentiation (2–4). Overexpression of Annexin A1 has been found in numerous cancer types (5,6). Annexin A2 induces angiogenesis by regulating the extracellular matrix metalloproteinase inducer. Furthermore, overexpression of Annexin A2 has been associated with the development, invasion and distant metastases of various tumors (7–11). Compared with other Annexins, few previous studies have investigated the function of Annexin A3. Köllermann *et al* previously reported that Annexin A3 staining was markedly decreased or absent in prostate cancer and was found to correlate inversely with pT stage and Gleason grade (12).

Yan *et al*(13) previously showed that the increased expression of Annexin A3 is a mechanism of platinum resistance in ovarian cancer. Similarly, the Annexin A3 gene is silenced by miRNA induced apoptosis and inhibits the growth of human gallbladder cancer cells (14). However, to date, a correlation between Annexin A3 expression and colorectal cancer has not been reported.

Hypoxia-inducible factor-1α (HIF-1α) has been found to correlate with the increased expression of vascular endothelial growth factor (VEGF) in various cancer types (15,16). The overexpression of HIF-1α has been associated with the poor prognosis of various types of cancer (17,18). Considering the important role of HIF-1α in the angiogenesis of tumors, HIF-1α is a target for cancer therapy. Similarly, Annexin A3 has been associated with the stimulation of VEGF expression and may be a regulator of angiogenesis. Park *et al*(19) found that Annexin A3-overexpressing human embryonic kidney (HEK) 293 cells induce the migration and tube formation of human umbilical vein endothelial cells. Furthermore, in HEK 293 cells, the expression of Annexin A3 activates HIF-1 transactivation activity, which indicates that Annexin A3 may regulate the HIF-1 signaling pathway. Therefore, Annexin A3 and HIF-1α have been hypothesized to be vital in the angiogenesis of cancer. However, no previous studies have investigated the correlation between Annexin A3 and HIF-1α expression in types of human cancer. In this study, the expression of Annexin A3 and HIF-1α was detected in colorectal cancer. The objective was to ascertain whether Annexin A3 is involved in the progression of colorectal cancer, and to investigate its correlation with HIF-1α. Additionally, the impact of Annexin A3 and HIF-1α on patient outcome was evaluated.

## Materials and methods

### Patients and tissues

A total of 60 unselected formalin-fixed and paraffin-embedded samples from colorectal cancer patients were obtained from the Department of Pathology, Xiangya Hospital of Central-South University (Changsha, China), between 1999 and 2001. All samples were diagnosed by two pathologists. Clinical and pathological results were investigated prospectively and the specific tumor registry was determined after surgery and follow-up. The follow-up deadline was December, 2010. Survival rate was calculated between the date of surgery and the last follow-up or date of mortality. Each case was classified according to the tumor size and localization, Dukes’ stage and differentiation degree. The study was approved by the ethics committee of Central-South University. Informed written consent was obtained from each patient.

### Immunohistochemistry

Annexin A3 and HIF-1α staining was performed using formalin-fixed and paraffin-embedded serial sections. Sections (4 μm) were cut from the selected paraffin blocks and deparaffinized by routine techniques. The slides were microwaved in citrate buffer for 6 min for antigen retrieval. Annexin A3 was detected using a rabbit polyclonal antibody (1:150) and HIF-1α was detected using a mouse polyclonal antibody (1:100; both Santa Cruz Biotechnology, Inc., Santa Cruz, CA, USA). The primary antibody reaction was performed at 4°C overnight. Labeling was detected by adding biotinylated secondary antibodies, avidin-biotin complex and diaminobenzidine (all Maxim-Bio, Fuzhou, China). Sections were then counterstained with hematoxylin. Staining results for each antibody were examined by two authors independently.

### Evaluation of Annexin A3 and HIF-1α expression

Annexin A3 and HIF-1α immunoreactivity was detected in the cytoplasm of tumor cells. The expression of Annexin A3 and HIF-1α was scored according to the positive percentage and staining intensity of the stained tumor cells. The percentage positivity was scored as follows: 0, 0–25%; 1, 26–50%; 2, 51–75%; and 3, >75%. The staining intensity was scored as follows: 0, no staining; 1, weak; 2, moderate; and 3, strong. If the product of multiplication between staining intensity and the percentage of positive cells was ≥2, it was considered as positive (+) immunoreactivity.

### Statistical analysis

Annexin A3 expression was assessed for a correlation with clinicopathological parameters and HIF-1α expression using the Fisher’s exact and Spearman’s correlation coefficient tests. For the survival analysis, the survival curves were calculated using the Kaplan-Meier method. All statistical analyses were performed using SPSS 11.0 for Windows (SPSS, Inc., Chicago, IL, USA). P<0.05 was considered to indicate a statistically significant difference.

## Results

### Correlation between Annexin A3 expression and clinicopathological variables

**Figure 1:**
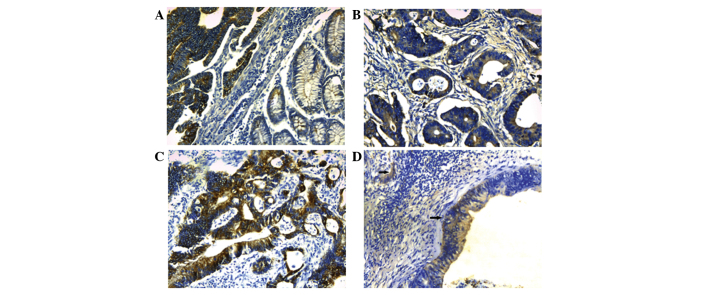
Immunohistochemical staining of Annexin A3 in colorectal cancer. (A) Staining for Annexin A3 protein was stronger in tumor (T) tissue than in adjacent noncancerous (N) tissue. Positive Annexin A3 staining in (B) well-differentiated and (C) moderately differentiated colorectal cancer and (D) lymph nodes, indicated by the arrows (labeled streptavidin-biotin staining; magnification, ×200).

Of the 60 colorectal cancer tissues, Annexin A3 staining was undetected in 21 cases. Compared with weak or no expression in normal colorectal tissues, 65% (39/60) of colorectal cancer specimens showed Annexin A3 immunoreactivity (Fig. 1). Annexin A3 was predominantly expressed in the cytoplasm of cancer cells. To improve the investigation of the role of Annexin A3 in colorectal cancer, the correlation between Annexin A3 expression and clinicopathological factors was analyzed. Tumor size and Dukes’ stage exhibited statistically significant correlations with Annexin A3 expression (Table I). However, no significant correlation was identified between the levels of Annexin A3 expression and other clinical and pathological features, including gender, age, tumor localization, tumor differentiation degree and lymph node metastasis.

### Correlation between HIF-1α expression and clinicopathological variables

HIF-1α expression was observed in 28 (47%) of the 60 cases. In positive cases, HIF-1α immunoreactivity was present in the cytoplasm of tumor cells. The correlation between HIF-1α and clinicopathological factors is exhibited in Table I. HIF-1α expression was found to significantly correlate with tumor size and differentiation degree. No significant correlation was identified between HIF-1α expression and the other clinicopathological factors, including gender, age, tumor localization, Dukes’ stage and lymph node metastasis.

### Correlation between Annexin A3 and HIF-1α expression

**Figure 2:**
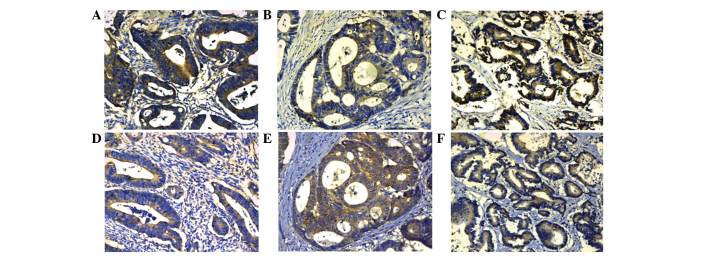
Immunohistochemical analysis of Annexin A3 and HIF-1α proteins in the same colorectal cancer samples. Positive staining for (A–C) Annexin A3 and (D–F) HIF-1α in the cytoplasm of colorectal cancer cells. HIF-1α, hypoxia-inducible factor-1α (labeled streptavidin-biotin staining; magnification, ×200).

Due to the important roles of Annexin A3 and HIF-1α in cancer progression, the correlation between Annexin A3 and HIF-1α expression was analyzed. In the same samples, Annexin A3 and HIF-1α exhibited similar expression patterns (Fig. 2). In total, 60 colorectal cancer samples were detected for Annexin A3 and HIF-1α staining. Of the 60 cases, 23 (38%) were positive for Annexin A3 and HIF-1α, whereas, Annexin A3 and HIF-1α were not expressed in 16/60 (27%) of cases. Furthermore, 16 of the 60 (27%) cases were Annexin A30-positive but HIF-1α-negative, whereas only HIF-1α was stained in 5/60 (8%) cases. A significant correlation was observed between Annexin A3 and HIF-1α expression (P=0.009; rs=0.336).

### Correlation between Annexin A3 expression and overall survival in patients with colorectal cancer

**Figure 3:**
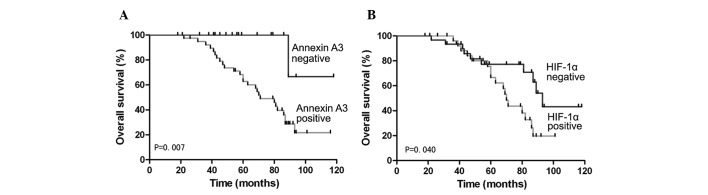
Kaplan-Meier analysis of overall survival curves of colorectal cancer patients, according to the expression of (A) Annexin A3 and (B) HIF-1α. HIF-1α, hypoxia-inducible factor-1α.

To investigate the prognostic role of Annexin A3 in colorectal cancer, overall survival rates were estimated by Kaplan-Meier survival. Within this group, patients with positive expression of Annexin A3 exhibited significantly shorter overall survival than patients negative for Annexin A3 (P=0.007) (Fig. 3). Similarly, overall survival in colorectal cancer patients was significantly different between patients positive for HIF-1α and patients negative for HIF-1α (P=0.040) (Fig. 3). Univariate and multivariate analyses were performed to evaluate the impact of Annexin A3 expression and pathological factors on the prognosis of colorectal cancer (Table II). Univariate Cox regression analysis indicated that tumor differentiation, Dukes’ stage, lymph node status and Annexin A3 expression were significantly associated with overall survival. In the multivariate analyses, Dukes’ stage, lymph node status and Annexin A3 expression were associated with poor overall survival. Annexin A3 expression levels are an indicator of a poor prognosis for overall survival (hazard ratio, 3.331; 95% CI, 1.213–9.098) (Table II). Of all the clinicopathological factors, Dukes’ stage was the most independent factor predicting prognosis (P=0.021).

## Discussion

Annexin A3 has been previously identified as an oncoprotein, based on its overexpression in lung adenocarcinoma and its ability to promote lymph node metastasis (20). However, it has been reported that Annexin A3 may play a different role in tumor development or progression among various human organs. Köllermann *et al* previously found that Annexin A3 protein expression was essentially reduced in prostate cancer and exhibited a negative correlation with the prognosis of patients (12). These contradictory studies led the present study to investigate the roles of Annexin A3 expression in colorectal cancer.

In the present study, a subset of the colorectal cancer cases with follow-up data were analyzed to show a correlation between the absence of Annexin A3 and clinical outcome. The results indicated that Annexin A3 protein expression was significantly increased in colorectal cancer tissues compared with the paired noncancerous tissues, which is consistent with the results in lung adenocarcinoma (20). These results indicate that Annexin A3 may act as an oncogene in colorectal cancer. Furthermore, overexpression of Annexin A3 was found to significantly correlate with tumor size and Dukes’ stage, which indicated that Annexin A3 expression may be important in the development of colorectal cancer. Furthermore, the high expression of Annexin A3 in colorectal cancer tissues was found to significantly correlate with shorter overall survival in patients, compared with that of low Annexin A3 expression. Cox regression analysis revealed that Dukes’ stage, lymph node metastases and Annexin A3 expression were independent prognostic factors in colorectal cancer patients. These results illustrate that Annexin A3 is a potentially important factor in the progression of colorectal cancer, as well as a novel molecular target of gene therapy for colorectal cancer.

Angiogenesis is strictly regulated via a balance of numerous angiogenic and anti-angiogenic factors. When the balance is disrupted, it leads to tumor development. It is known that HIF-1α is a major factor in the regulation of VEGF expression (16). Previously, Park *et al*(19) found that Annexin A3 may affect vascular formation by directly or indirectly regulating VEGF expression. Furthermore, luciferase assay results show that Annexin A3 activates HIF-1 activity in HEK 293 cells and HIF-1 may be a target of Annexin A3. Consistent with these observations, the current study also found a close correlation between Annexin A3 and HIF-1α expression in colorectal cancer tissues. The results indicate that Annexin A3 may be involved in tumor angiogenesis by regulating HIF-1α. Future studies are required to elucidate the correlation between Annexin A3 and HIF-1α. In addition, HIF-1α expression has been found to correlate significantly with tumor size and differentiation. However, in multivariate Cox model analysis, HIF-1α was not an independent prognostic factor for the overall survival of colorectal cancer patients.

In conclusion, the present study is the first to evaluate Annexin A3 expression and its correlation with clinicopathological factors in colorectal cancer tissues. The results indicate that the increased expression of Annexin A3 in colorectal cancer significantly correlates with tumor progression and poor prognosis. In addition, Annexin A3 and HIF-1α proteins appear to be important in the advancement of colorectal cancer. It was also found that Annexin A3 expression is associated with HIF-1α expression. These observations highlight an improved understanding of the carcinogenesis of colorectal cancer and Annexin A3 may be a potential biomarker for predicting the prognosis of colorectal cancer patients.

## Figures and Tables

**Table I:** Correlation between Annexin A3 or HIF-1α expression and clinicopathological features of the colorectal cancer patients.

	Annexin A3 expression			HIF-1α expression		
				
Variable	Negative	Positive	P-value	Negative	Positive	P-value
Gender						
Male	15	25		19	21	0.274
Female	6	14	0.775	13	7	
Age, years						
≤60	11	18		17	12	0.451
>60	10	21	0.788	15	16	
Tumor size, cm						
≤2	13	11		18	6	0.008
>2	8	28	0.014	14	22	
Tumor localization						
Right colon	7	15		8	14	0.062
Left colon	14	24	0.783	24	14	
Tumor differentiation						
Grade 1	4	10		11	3	0.037
Grades 2 and 3	17	29	0.751	21	25	
Dukes’ stage						
A+B	15	11		13	13	0.795
C+D	6	28	0.002	19	15	
Lymph node metastasis						
No	13	20		21	12	0.118
Yes	8	19	0.587	11	16	

HIF-1α, hypoxia-inducible factor-1α.

**Table II:** Univariate and multivariate analysis of overall survival in patients with colorectal cancer.

	Univariate analysis			Multivariate analysis		
		
Variable	Hazard ratio	95% CI	P-value	Hazard ratio	95% CI	P-value
Gender	1.678	0.742–4.126	0.268			
Age, years	1.012	0.971–1.055	0.560			
Tumor localization	0.886	0.264–2.971	0.844			
Tumor differentiation	5.641	2.278–12.981	0.000	2.627	0.897–6.154	0.059
Tumor size, cm	1.001	0.325–3.084	0.999			
Dukes’ stage	0.117	0.021–0.448	0.002	0.188	0.037–0.861	0.021
Lymph node status	6.896	2.405–20.863	0.001	3.586	1.056–10.231	0.029
HIF-1α expression	1.552	0.600–4.015	0.365			
Annexin A3 expression	6.875	2.471–20.057	0.001	3.331	1.213–9.098	0.031

HIF-1α, hypoxia-inducible factor-1α.
